# Identification and Expression Analysis of the Interferon-Induced Protein with Tetratricopeptide Repeats 5 (*IFIT5*) Gene in Duck (*Anas platyrhynchos domesticus*)

**DOI:** 10.1371/journal.pone.0121065

**Published:** 2015-03-27

**Authors:** Bin Wang, Yang Chen, Chunyu Mu, Yanhui Su, Ran Liu, Zhengyang Huang, Yang Li, Qingming Yu, Guobin Chang, Qi Xu, Guohong Chen

**Affiliations:** Key Laboratory of Animal Genetics & Breeding and Molecular Design of Jiangsu province, Yangzhou University, Yangzhou, People's Republic of China; CSIRO, AUSTRALIA

## Abstract

The interferon-induced proteins with tetratricopeptide repeats (IFITs) protein family mediates antiviral effects by inhibiting translation initiation, cell proliferation, and migration in the interferon (IFN) dependent innate immune system. Several members of this family, including *IFIT1*, *IFIT2*, *IFIT3* and *IFIT5*, have been heavily studied in mammals. Avian species contain only one family member, *IFIT5*, and little is known about the role of this protein in birds. In this study, duck *IFIT5* (*duIFIT5*) full-length mRNA was cloned by reverse transcription polymerase chain reaction (RT-PCR) and rapid amplification of the cDNA ends (RACE). Based on the sequence obtained, we performed a series of bioinformatics analyses, and found that *duIFIT5* was most similar to homologs in other avian species. Also, *duIFIT5* contained eight conserved TPR motifs and two conserved multi-domains (TPR_11 and TPR_12). Finally, we used duck hepatitis virus type 1 (DHV-1) and polyriboinosinicpolyribocytidylic acid (poly (I:C)) as a pathogen or a pathogen-associated molecular pattern induction to infect three-day-old domestic ducklings. The liver and spleen were collected to detect the change in *duIFIT5* transcript level upon infection by quantitative real-time PCR (qRT-PCR). *DuIFIT5* expression rapidly increased after DHV-1 infection and maintained a high level, while the transcripts of *duIFIT5* peaked at 8h after poly (I:C) infection and then returned to normal. Taken together, these results provide a greater understanding of avian *IFIT5*.

## Introduction

The innate immune system relies on a class of cytokines known as interferons (IFNs), which are secreted by host cells in response to viral infection [1,2]. IFNs, combined with specific cell surface receptors, lead to high expression of a large group of IFN-stimulated genes (ISGs), which inhibit viral replication, transcription and cell proliferation [3,4].

IFITs are among the most predominant ISGs, and based on extensive studies from recent years, have been shown to play a significant role during viral infection [5]. Four members, *IFIT1* (p56; ISG56), *IFIT2* (p54; ISG54), *IFIT3* (p60; ISG60), and *IFIT5* (p58; ISG58), constitute an intimate family in mammals. IFITs are conserved in mammals, amphibians and fish, but do not exist in lower animals. *IFIT5* is the sole family member found in birds. Very little is known about the function of *IFIT5* in birds.

IFITs are characterized by multiple tetratricopeptide repeats (TPRs), degenerate helix–turn–helix motifs of 34 amino acids in length. Different IFIT family members have distinct numbers of TPR motifs, for example, *IFIT1* has six TPR motifs, while *IFIT5* has eight. Over the past decade, the antiviral activity and mechanisms of action of IFIT proteins have been gradually elucidated. Recently, IFIT family members have been shown to selectively restrict viral replication by sensing the methylation status of the 5’-cap of some viral RNAs [6–8]. Viruses hijack a cap from host mRNA or encode machinery to add a 5’-cap structure to their mRNA, thereby escaping IFIT recognition [9]. More recently, the structural basis for viral 5’-PPP-RNA recognition by human IFIT proteins has been unraveled, which validated the mechanism by which IFIT proteins selectively recognize viral single-stranded RNA (ssRNA) [10].

In this study, we presented the molecular cloning and characterization of *duIFIT5* and analyzed its expression during duck hepatitis virus type 1 (DHV-1) and poly (I:C) infection, to investigate infection by a single-stranded RNA (ssRNA) and a double-stranded RNA (dsRNA), respectively. These data facilitated a better understanding of the role of *duIFIT5* in immunity, thus providing tools for future immunopathological studies.

## Materials and Methods

### Ethics Statement

All animal experiments were reviewed and approved by the Institutional Animal Care and Use Committee of Yangzhou University. Experiments were performed in accordance with the Regulations for the Administration of Affairs Concerning Experimental Animals (Yangzhou University, China, 2012) and the Standards for the Administration of Experimental Practices (Jiangsu, China, 2008). All operations were performed according to recommendations proposed by the European Commission (1997), and all efforts were made to minimize suffering.

### Animals and sample collection

All animals were obtained from the Chinese Waterfowl Germplasm Resource Pool (Taizhou, China). To characterize gene expression, various tissues, including heart, liver, spleen, lung, kidney, large intestine, small intestine, muscle, cerebrum, cerebellum, glandular stomach and muscular stomach, were collected from healthy, adult, domestic ducks, frozen in liquid nitrogen, and stored at −80°C.

120 three-day-old domestic ducklings were randomly assigned to three groups and injected with 0.4 mL of either allantoic fluid containing DHV-1, poly (I:C) (0.5 mg/mL, Invitrogen, USA), or saline (as a control). Ducklings inoculated with DHV-1 appeared depressed soon, showed little desire to eat, and began to die 1.5 d.p.i, whereas the forty ducklings inoculated with poly (I:C) and saline have no symptoms. Samples from ducklings with no symptoms were collected at 0, 4, 8, 12, 24, 36, 48, 72, and 96 h after injection. 4 ducklings per group were euthanized after anesthesia with intraperitoneal injection of sodium pentobarbital (150mg/kg) at each time point. The spleen and liver were snap-frozen in liquid nitrogen immediately after dissection and stored at -80°C. Ducklings displayed symptoms were anaesthetic by using a low dose of sodium pentobarbital (50mg/kg). If ducklings displayed serious symptoms and seemed moribund, they would be euthanized by using a high dose of sodium pentobarbital (150mg/kg) and decollation.

### RNA extraction and cDNA synthesis

Total RNA was extracted using Trizol reagent (Takara) according to the standard protocol. 1 μg of RNA isolated from the tissues was used to synthesize first strand cDNA with the cDNA synthesis kit (Takara) according to the manufacturer’s protocol.

### Cloning of *duIFIT5*

The TaKaRa 5’-Full RACE Kit and 3’-Full RACE Core Set Ver.2.0 were used according to the manufacturer’s instructions to amplify the 5’ and 3’ ends of the cDNA, respectively. RACE primers (S1 Table) were designed using the *duIFIT5* CDS obtained from RT-PCR. Touchdown and nested PCRs were performed according to the manufacturer’s instructions. Amplication were then cloned into a plasmid vector for nucleotide sequencing, as described above.

### Bioinformatic analysis

BLASTn (http://blast.ncbi.nlm.nih.gov/Blast.cgi) was used to identify genes with homology to *IFIT5*. Homology analyses of nucleotide and amino acid sequences were performed with DNAstar software. A phylogenetic tree was generated by the neighbor joining method with 1000 bootstrap replicates in MEGA5.0. ESPript3.0 (http://espript.ibcp.fr/ESPript/cgi-bin/ESPript.cgi) was used to construct multiple alignments of the amino acid sequences of *IFIT5* proteins. Conserved domains within *duIFIT5* and human *IFIT5* were identified through NCBI (http://www.ncbi.nlm.nih.gov/Structure/cdd/wrpsb.cgi). The primary structure and subcellular localization was analyzed using the ExPASy ProtParam tool (http://espript.ibcp.fr/ESPript/cgi-bin/ESPript.cgi) and PSORT II Protein Sorting Prediction program (http://psort.ims.u-tokyo.ac.jp/form2.html).

### Quantitative real-time PCR (qRT-PCR)

To quantify *duIFIT5* gene expression patterns, cDNA from different tissues was amplified in a 20 μL reaction using the Applied Biosystems 7500 real-time PCR system with the following program: 1 cycle at 95°C for 30 s, followed by 40 cycles of 95°C for 5 s and 60°C for 34 s. All cDNA samples were tested three times, and the results were normalized to duck glyceraldehyde-3-phosphate dehydrogenase (*GAPDH*) expression. The primers designed for real-time PCR (P7 and P8 for *duIFIT5*; P9 and P10 for *GAPDH*) are shown in S1 Table. The relative expression levels of *duIFIT5* in healthy and infected ducks were indicated by the 2^− ΔCt^ and 2^− ΔΔCt^ methods, respectively.

### Construction of plasmids and transient transfection

The CDS of *duIFIT5* (P1 and P2) was cloned into the EGFP-C1 vector (Invitrogen, USA) digested with *XbaI* and *KpnI* to produce the EGFP-C1-*duIFIT5* plasmid. DF1 cells were seeded at 2×10^5^ cells/well (24-well plate), grown to 80% confluence, and then transfected with 2 μL Lipofectamine-2000 transfection reagent (Invitrogen, USA) and 1μg of plasmid DNA (EGFP-C1-*duIFIT5* or EGFP-C1) according to the manufacturer’s instructions. The complexes were removed after 6 h, and the complete growth medium was replaced. Finally, fluorescence microscopy was used to detect the fluorescence in each well at 24 h post-transfection.

## Results

### cDNA cloning of *duIFIT5* and bioinformatics analysis

The full-length cDNA sequence of *duIFIT5* was 2146 bp in size, including a 71 bp 5′ UTR and a 635 bp 3′ UTR with the poly [A] tail (Genbank accession No.KF956064). The open reading frame (ORF) was 1440 bp in length and encoded a predicted protein of 479 amino acids. The nucleotide and predicted amino acid sequences of the *duIFIT5* gene are shown are shown in S1 Fig.

**Fig 1:**
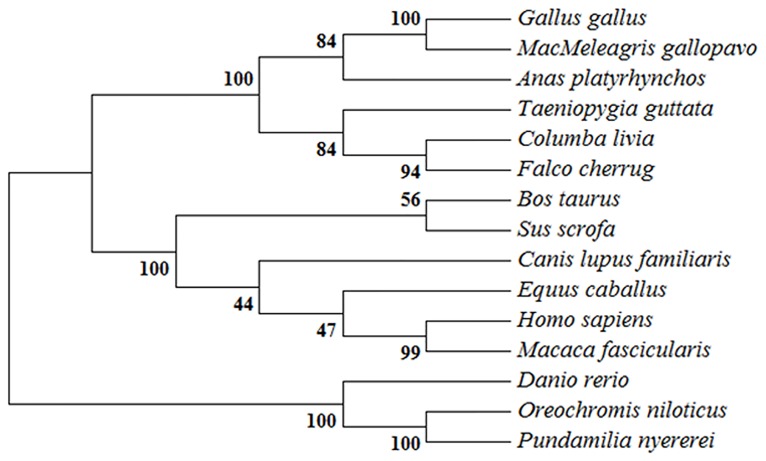
Phylogenetic tree of *IFIT5* amino acid sequences generated with the neighbor-joining tree method. Numbers at each branch indicate the percent a node was supported in 1,000 bootstrap replicates.

**Fig 2:**
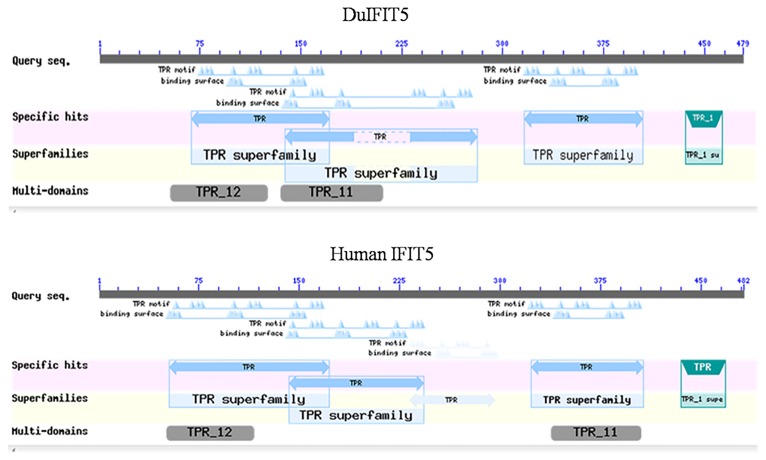
The predicted conserved domains predicted from the amino acid sequence of *duIFIT5* and human *IFIT5*. Both *duIFIT5*and human *IFIT5* have eight TPR motifs and multi-domains TPR_11 and TPR_12.

DNAStar software was used to analyze *duIFIT5*. Compared to the homologous gene sequences of nine other species obtained from Genbank, it had the highest homology to *Falco cherrug* (up to 80.1%), followed by *Meleagris gallopavo* (up to79.7%), and about 50% homology with other species (S2 Fig.). A condensed phylogenetic tree was constructed based on the amino acid sequence of *duIFIT5* compared to homologs in other organisms (Fig. 1). The overall topology of the tree revealed three major groups, including mammals, birds, and fish. *DuIFIT5* was most similar to homologs of the other avian species. Aligning the amino acid sequences of *duIFIT5* homologs from several species using ESPript3.0 software, we found that the amino acid sequences were conserved between mammals, fish, and birds (S3 Fig.). The conserved domains predicted from the amino acid sequence included eight TPR modifs and two multi-domains (TPR_11 and TPR_12), which is similar to the domain structure of human *IFIT5* (Fig. 2). The ExPASyProtParam and PSORT Protein Sorting Prediction tools were used to analyze the primary structure and subcellular localization of *duIFIT5*. Primary structure analysis showed that the predicted molecular weight was 55780.8 Da with a theoretical isoelectric point of 6.57. Subcellular localization analysis predicted that *duIFIT5* would be highly expressed in both the cytoplasm and nucleus (S2 Table).

### Subcellular localization of *duIFIT5* in DF1 cells

**Fig 3:**
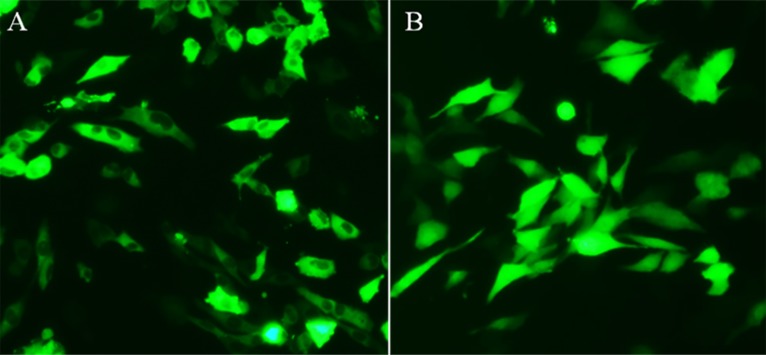
Subcellular localization of *duIFIT5* in DF1 cells. EGFP-C1-*duIFIT5* or an EGFP-C1 control plasmid was transiently transfected into DF1 cells, shown in A and B, respectively. *DuIFIT5* was mostly in the cytoplasmic, but was also detected in the nucleus.

To confirm the above prediction, the EGFP-C1-*duIFIT5* plasmid, or EGFP-C1 as a negative control, was transfected into chicken embryo fibroblasts (DF1 cells), and fluorescence was observed 24 h post-transfection (Fig. 3). *DuIFIT5* was predominantly in the cytoplasmic, but was also visualized in the nucleus.

### Tissue expression of *duIFIT5* in healthy ducks

**Fig 4:**
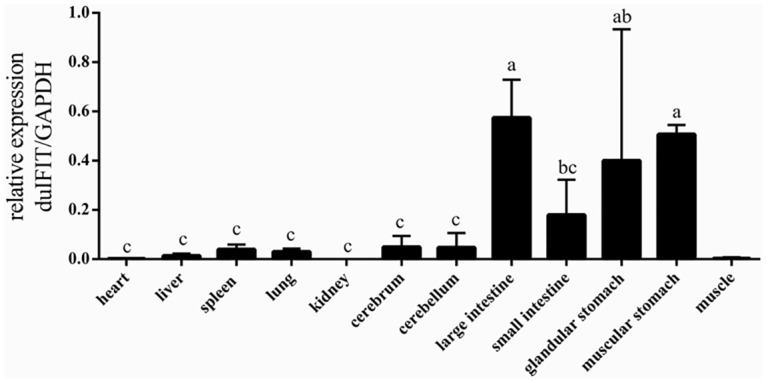
Relative expression levels of *duIFIT5* in the following tissues: heart, liver, spleen, lung, kidney, cerebrum, cerebellum, large intestine, small intestine, glandular stomach, muscular stomach, and muscle. The expression of *duIFIT5* was normalized to *GAPDH*. Different letter showed significant difference (p < 0.05).

To determine which tissues expressed *duIFIT5*, qRT-PCR was performed using gene specific primers and cDNA templates synthesized from several tissues taken from healthy ducks. *DuIFIT5* mRNA was constitutively expressed in all 12 tissues tested. The highest expression levels were observed in intestine (large intestine and small intestine) and stomach (glandular stomach and muscular stomach) tissues, whereas the expression levels in other tissues were relatively low, especially in heart, kidney and muscle. (Fig. 4).

### Temporal expression of *duIFIT5* after DHV-1 and poly (I:C) injection

**Fig 5:**
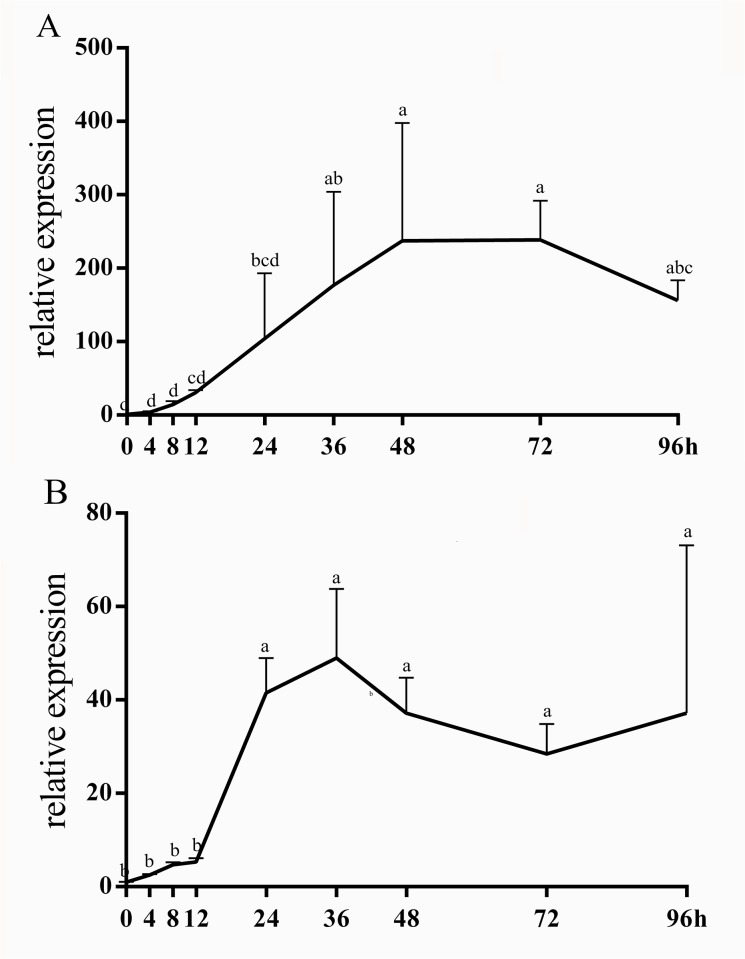
Relative expression of *duIFIT5* in liver (A) and spleen (B) after DHV-1 injection. qRT-PCR was used to determine the relative expression of *duIFIT5* in liver and spleen tissues at 0, 4, 8, 12, 24, 36, 48, 72 and 96 h after infection with DHV-1. The expression of *duIFIT5* was normalized to *GAPDH*. Different letter showed significant difference (p < 0.05).

**Fig 6:**
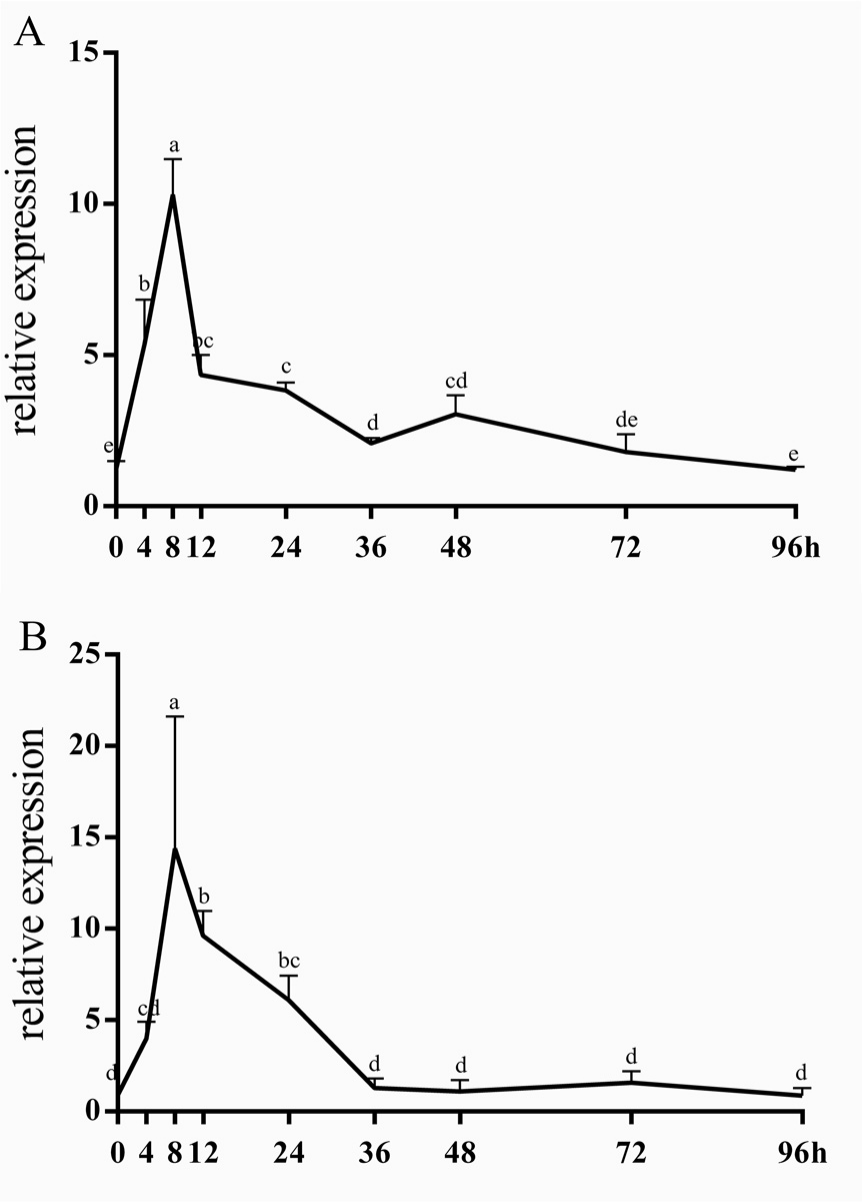
Relative expression of *duIFIT5* in liver (A) and spleen (B) after poly (I:C) injection. qRT-PCR was used to determine the relative expression of *duIFIT5* in liver and spleen tissues at 0, 4, 8, 12, 24, 36, 48, 72 and 96 h after infection with poly (I:C). The expression of *duIFIT5* was normalized to *GAPDH*. Different letter showed significant difference (p < 0.05).

Upon infection of ducklings with DHV-1, the major pathological change is hepatitis. The same situation also appeared in the dead ducklings of our experiment. In addition, the spleen is an important organ for immune system function. Therefore, liver and spleen tissues of treated and untreated groups were collected to study the temporal expression of *duIFIT5* with injection of foreign RNA. The mRNA expression levels of *duIFIT5* in liver and spleen were measured at various time points after injection (0, 4, 8, 12, 24, 36, 48, 72, 96 h) by qRT-PCR. For animals infected with DHV-1, some subtle differences were observed in the expression patterns of the spleen compared to the liver. In the liver, *duIFIT5* expression sharply increased more than 200- fold from 0–48 hours post-infection and expression levels remained high compared with that of the control for the duration of the experiment. In the spleen, *duIFIT5* expression peaked at 36 h post-infection, but only demonstrated a 40-fold increase with that of the control (Fig. 5). After poly (I:C) injection, *duIFIT5* expression rapidly increased in both liver and spleen during the first eight hours post infection and reached a peak of 10–20 fold with that of that control, then returned to normal levels by 36 h post induction (Fig. 6). These results showed that *duIFIT5* sharply increased following both types of viral infection, but the temporal expression patterns of DHV-1 group and poly (I:C) group were different. The expression pattern of DHV-1group was similar between the liver and spleen, but *duIFIT5* shown a stronger increase in liver.

## Discussion

We cloned the 2146-bp mRNA of *duIFIT5*, performed bioinformatics analysis, and determined the subcellular localization of *duIFIT5* in DF1 cells. We found *duIFIT5* was closely related to homologs found in turkey and chicken. Aligning the amino acid sequences of *duIFIT5* homologs from several species, we found that the domains were conserved between mammals, fish, and birds. The analyses provided an evidence that the IFIT family is evolutionarily conserved from mammals to birds. Besides, the conserved domains of *duIFIT5* consisted of eight TPR motifs and two multi-domains (TPR_11 and TPR_12), which was similar to the domain structure of human *IFIT5*. The presence of tandem arrays of multiple TPR domains provides a well-suited space for mediating protein–protein interactions, potentially allowing IFIT proteins to bind to specific proteins and RNAs, regulating the cell cycle[11–13]. Recently, the crystal structure of IFIT protein has been elucidated, revealing that helix-turn-helix TPR-like structures exist in each subunit, forming a nucleotide channel which binds specific proteins and RNAs[14,15]. This prompted us *duIFIT5* may have a similar fuction as human *IFIT5*.

Although IFITs are normally quiescent, their transcription is strongly induced by IFNs, virus infection, and molecular patterns such as double-stranded RNA or lipopolysaccharides [16,17]. We have demonstrated that this is coincident for *duIFIT5*. The relative expression level of *duIFIT5* compared to *GAPDH* is very low in most tissues, except in the intestine and the stomach. The high expression in these tissues may be attributed to unknown microbes in these organs, such as viruses, bacteria, and fungi, which potentially trigger induction of IFNs [18,19].

Following we decided to mimic foreign RNA viral infection in vivo to determine the temporal expression of *duIFIT5* after DHV-1 and poly (I:C) infection. The challenging ducklings experiment with DHV-1 and poly (I:C) infection resulted in two different *duIFIT5* temporal expression patterns. There was a strong increase in *duIFIT5* expression during both types of viral infection, however, the experiment revealed a different expression tendency to DHV-1and poly (I:C). In DHV-1 group, *duIFIT5* expression sharply increased to a peak and maintained high. In Poly (I:C) group, *duIFIT5* expression increased to a peak and rapidly returned to the normal. This phenomenon may be caused by ducklings’ different resistance to the two viruses. DHV-1 is an acute and fatal disease of young ducklings. It is very prevalent and results in mortality rates higher than 90% in infected ducklings under 3 weeks of age [20–22]. As *IFN-α*transcripts greatly increase following DHV-1 infection[23], the elevated expression of *duIFIT5* was expected in our study. And the recent hypothesis that IFIT5 specifically engage single-stranded 5’-PPP-RNA could also explain why abundant transcripts of *duIFIT5* persisted in both liver and spleen. The major pathologic change in infected ducklings is hepatitis, hence, the exceptionally higher expression level detected in the liver may be due to higher pressure against numerous viruses. Similar *IFIT5* expression changes have been observed upon avian influenza infection. Several papers have shown that *IFIT5* is very highly expressed in avian influenza infected ducklings [24,25]. Poly (I:C) is a synthetic double-stranded RNA that has been identified as a product of viral replication [26]. Previous studies have demonstrated that IFN-β and ISGs were over expressed by host cells after poly (I:C) infection [27]. Our result indicates that upon poly (I:C) infection *duIFIT5* obviously increased. Poly (I:C) is nontoxic or lentogenic to duck, so the rapid return to basal level of *duIFIT5* expression might be because ducklings have a relatively high resistance to poly (I:C). Besides, our previous study have shown that duck RIG-I gene had a similar expression pattern after poly (I:C) infection[28]. *RIG-I* is considered to be a upstream gene for IFITs[29,30], so the result could also demonstrate that duck *RIG-I* and *IFIT5* are well connected. Together, our study revealed *duIFIT5* take part in both ssRNA and dsRNA innate immune process, and two different expression patterns of *duIFIT5* are put forth. These results provide a better understanding of *IFIT5* in avian species.

## Supporting Information

S1 Fig Nucleotide and predicted amino acid sequences of *duIFIT5*. (TIF)

S2 Fig Homology comparison of *IFIT5* amino acid sequences in duck and other vertebrates. The columns and rows show numbers representing each species. The intersection between a row and column shows the amino acid homology of *IFIT5* for the two corresponding species.
(TIF)

S3 Fig Amino acid alignment of *IFIT5* homologs. The alignment of primary and secondary structure of *IFIT5* proteins from ten species, *Homo sapiens* (Homo), *Gallus gallus* (Gallus), *Meleagris gallopavo* (Meleagris), *Anas platyrhynchosv* (Anas), *Sus scrofa* (Sus), *Bos Taurus* (Bos), *Poephila guttata* (Poephila), *Macaca fascicularis* (Macaca), *Equus caballus* (Equus), *Oreochromis niloticus* (Oreochromis) was shown. Ten species of *IFIT5* proteins have similar amino acid sequences.
(TIF)

S1 Table Primers used in this study. (DOCX)

S2 Table Prediction of *duIFIT5* subcellular localization. (DOCX)
