# Supplementary figures and images for: Identification and Expression Analysis of the Interferon-Induced Protein with Tetratricopeptide Repeats 5 (IFIT5) Gene in Duck (Anas platyrhynchos domesticus)
Source: PLoS One. 2015 Mar 27;10(3):e0121065. doi: 10.1371/journal.pone.0121065 (PMC4376821; doi:10.1371/journal.pone.0121065)

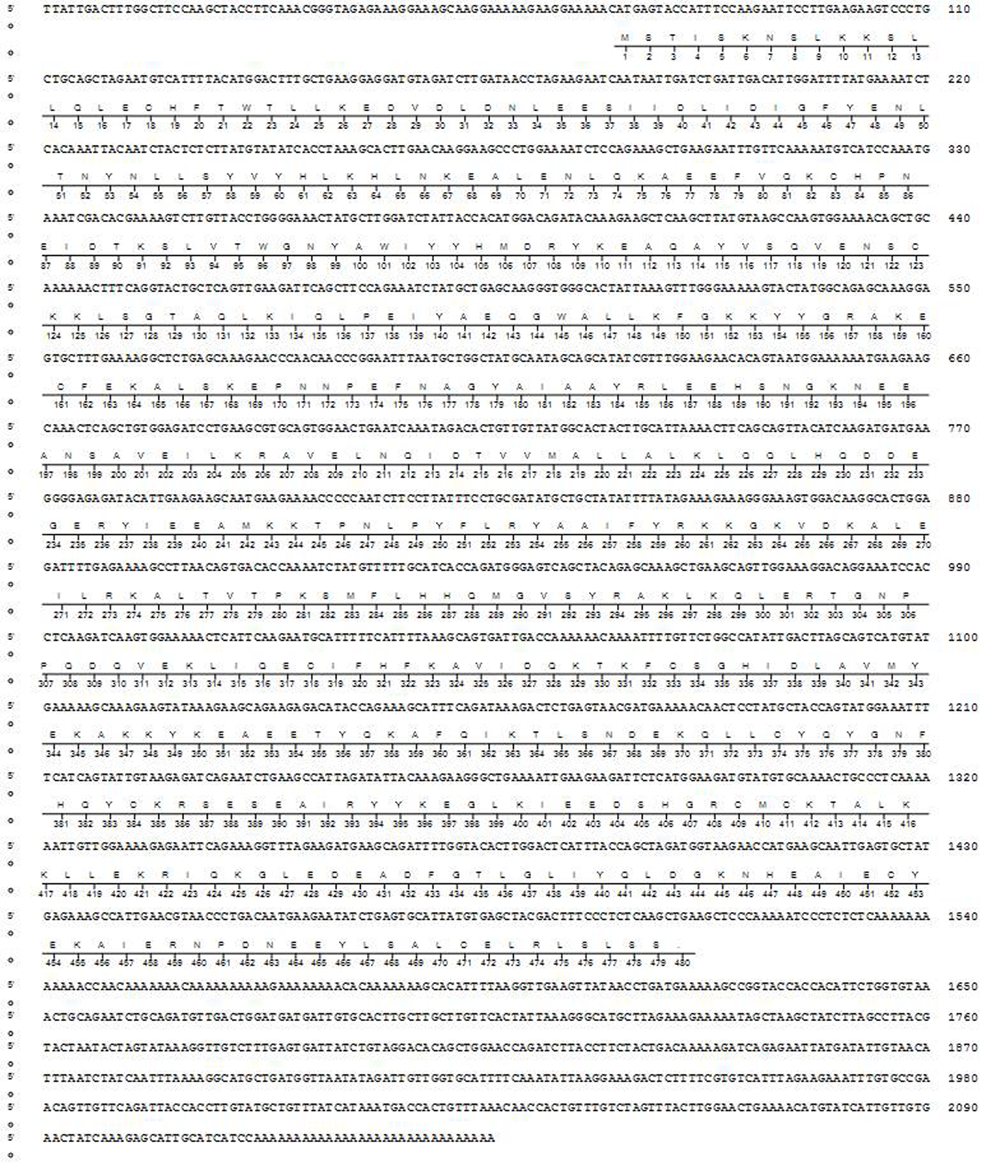

Supplement: S1 Fig — (TIF) [file pone.0121065.s001.tif]

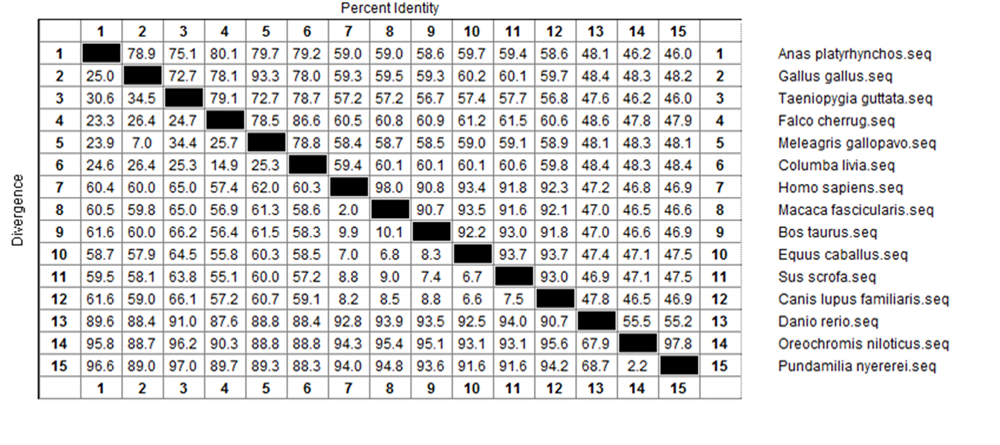

Supplement: S2 Fig — The columns and rows show numbers representing each species. The intersection between a row and column shows the amino acid homology of IFIT5 for the two corresponding species. (TIF) [file pone.0121065.s002.tif]

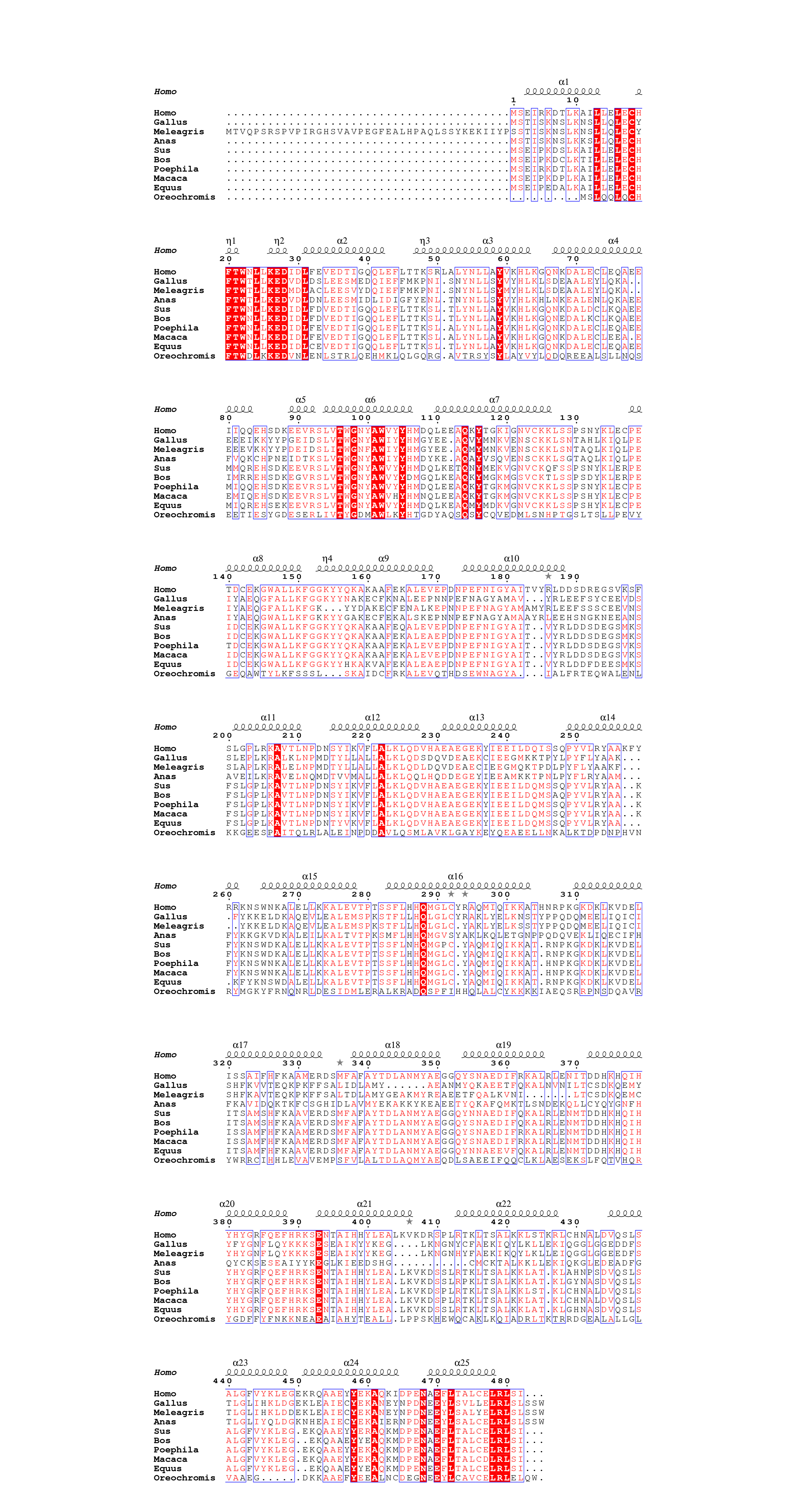

Supplement: S3 Fig — The alignment of primary and secondary structure of IFIT5 proteins from ten species, Homo sapiens (Homo), Gallus gallus (Gallus), Meleagris gallopavo (Meleagris), Anas platyrhynchosv (Anas), Sus scrofa (Sus), Bos Taurus (Bos), Poephila guttata (Poephila), Macaca fascicularis (Macaca), Equus caballus (Equus), Oreochromis niloticus (Oreochromis) was shown. Ten species of IFIT5 proteins have similar amino acid sequences. (TIF) [file pone.0121065.s003.tif]
