# Supplementary material for: Identification and Expression Analysis of the Interferon-Induced Protein with Tetratricopeptide Repeats 5 (IFIT5) Gene in Duck (Anas platyrhynchos domesticus)
Source: PLoS One. 2015 Mar 27;10(3):e0121065. doi: 10.1371/journal.pone.0121065 (PMC4376821; doi:10.1371/journal.pone.0121065)
Supplement: S1 Table — (DOCX) [file pone.0121065.s004.docx]

Table S1: Primers used in this study.

| **Primer ID** | **Primer Name** | **Sequence (5′→3′)** | **Annealing temperature (°C)** | **Application** |  |  |  |  |  |
| --- | --- | --- | --- | --- | --- | --- | --- | --- | --- |
| P1  P2 | IFIT5-F  IFIT5-R | CCG*CTCGAG*ATGAGTACCATTTCCAAGAATTCCTCGG*GGTACC***TCA**GCTTGAGAGGGAAAGTC | 61 | CDS amplification and vector construction |  |  |  |  |  |
| P3  P4 | 5’RACE Outer  5’RACE Inner | GGCCACGCGTCGACTAGTACGGGGGGGGGGGGGGGG  ATGGACTTTGCTGAAGGAGG | 60 | 5’RACE |  |  |  |  |  |
| P5  P6 | 3’RACE Outer  3’RACE Inner | ACAGGCTGCTCATTGCTACATCT  ACCCTGACAATGAAGAATATCTGAGTGC | 60 | 3’RACE |  |  |  |  |  |
| P7  P8 | Duck-IFIT5-F  Duck-IFIT5-R | AAGCTACCTTCAAACGGGTA  TCCTCCTTCAGCAAAGTCCA | 60 | RT-qPCR |  |  |  |  |  |
| P9  P10 | Duck-GAPDH-F  Duck-GAPDH-R | TGCTAAGCGTGTCATCATCT  AGTGGTCATAAGACCCTCCA | 60 | RT-qPCR |  |  |  |  |  |

NOTE: The underlined italics indicate the restriction enzyme recognition sites and the bold letters are the termination codon.
