# Supplementary material for: Identification and Expression Analysis of the Interferon-Induced Protein with Tetratricopeptide Repeats 5 (IFIT5) Gene in Duck (Anas platyrhynchos domesticus)
Source: PLoS One. 2015 Mar 27;10(3):e0121065. doi: 10.1371/journal.pone.0121065 (PMC4376821; doi:10.1371/journal.pone.0121065)
Supplement: S2 Table — (DOCX) [file pone.0121065.s005.docx]

Table S2: Prediction of *duIFIT5* subcellular localization

| **Percent** | **Location** |
| --- | --- |
| 39.1 % | Cytoplasmic |
| 34.8 % | Nuclear |
| 13.0 % | Cytoskeletal |
| 4.3 % | Vacuolar |
